# Anisotropic Optical Response of Ti-Doped VO_2_ Single Crystals

**DOI:** 10.3390/ma17133121

**Published:** 2024-06-25

**Authors:** Salvatore Macis, Lorenzo Mosesso, Annalisa D’Arco, Andrea Perucchi, Paola Di Pietro, Stefano Lupi

**Affiliations:** 1 Department of Physics, Sapienza University, Piazzale Aldo Moro 5, 00185 Rome, Italy; lorenzo.mosesso@uniroma1.it (L.M.); stefano.lupi@roma1.infn.it (S.L.); 2 Elettra—Sincrotrone Trieste S.C.p.A, S.S. 14 km163.5 in AREA Science Park, 34012 Trieste, Italy

**Keywords:** vanadium oxide, VO_2_, Ti doping, anisotropy, infrared spectroscopy

## Abstract

This study delves into the effects of titanium (Ti) doping on the optical properties of vanadium dioxide (VO_2_), a material well known for its metal–to–insulator transition (MIT) near room temperature. By incorporating Ti into VO_2_’s crystal lattice, we aim to uncover the resultant changes in its physical properties, crucial for enhancing its application in smart devices. Utilizing polarized infrared micro–spectroscopy, we examined Ti_*x*_V_1−*x*_O_2_ single crystals with varying Ti concentrations (x = 0.059, x = 0.082, and x = 0.187) across different crystal phases (the conductive rutile phase and insulating monoclinic phases M1 and M2) from the far–infrared to the visible spectral range. Our findings reveal that Ti doping significantly influences the phononic spectra, introducing absorption peaks not attributed to pure VO_2_ or TiO_2_. This is especially notable with polarization along the crystal growth axis, mainly in the x = 0.187 sample. Furthermore, we demonstrate that the electronic contribution to optical conductivity in the metallic phase exhibits strong anisotropy, higher along the c axis than the a–b plane. This anisotropy, coupled with the progressive broadening of the zone center infrared active phonon modes with increasing doping, highlights the complex interplay between structural and electronic dynamics in doped VO_2_. Our results underscore the potential of Ti doping in fine-tuning VO_2_’s electronic and thermochromic properties, paving the way for its enhanced application in optoelectronic devices and technologies.

## 1. Introduction

Since the discovery of the metal–to–insulator transition (MIT) phenomena in vanadium dioxide (VO_2_) [1], this material has received immense interest in the scientific community. Above the temperature of 341 K, VO_2_ undergoes a unique phase transition, transforming from an insulating monoclinic structure (P21/c, called M1) to a metallic state [1,2]. This transition is accompanied by substantial changes in its electrical conductivity, optical properties, and thermal response [1,2,3]. Many efforts have been made to understand whether the transition has an electronic origin (Mott–Hubbard transition) or if it is driven by structural distortion (Peierls transition) and to investigate its possibilities in optoelectronic devices [4,5,6]. These intriguing characteristics have positioned VO_2_, together with other vanadium oxides, as promising materials for a wide range of applications, including smart windows, thermochromic devices, energy–efficient coatings, and thermal sensors [5,6,7,8,9,10,11,12,13,14,15,16,17]. However, despite VO_2_ exhibiting remarkable properties, research is focused on enhancing its properties and expanding its application potential by incorporating dopants into its crystal lattice. Among these dopants, titanium (Ti) has emerged as a prominent candidate due to its compatibility with the VO_2_ lattice structure and its general ability to induce significant changes in materials’ properties [18,19,20]. One of the key advantages of Ti doping in VO_2_ lies in its ability to fine–tune the critical temperature at which the metal–insulator transition occurs [18]. By carefully controlling the doping concentration, it is possible to shift the transition temperature. This tunability allows for the design and optimization of VO_2_-based devices with enhanced performance and adaptability. Moreover, Ti doping influences other crucial properties of VO_2_, such as electrical conductivity and optical characteristics, by inducing changes in the electronic band structure due to lattice distortions [7,18,19]. One of the results of light titanium doping is the appearance of a second insulating monoclinic phase called M2, which has been obtained with small uniaxial [7] stress applied to pure VO_2_ samples or substituting vanadium with other transition metal ions in lower oxidation states. This work describes a spectroscopic investigation performed on Ti_*x*_$V_{1-x}$O_2_ single crystals, with titanium concentrations x = 0.059, x = 0.082, and x = 0.187 [18]. We show how the presence of titanium affects the phononic spectra of the monoclinic phases and determines a group of absorption peaks that are not simply related to pure vanadium dioxide or pure titanium dioxide. Moreover, we discuss the electronic contribution to the optical conductivity in the metallic phase R, which is strongly anisotropic and is weakened with growing titanium concentration.

## 2. Materials and Methods

Ti_*x*_V_1−*x*_O_2_ single crystals were grown using a high–temperature solution technique, which guarantees that the samples are as free from strain as possible [18]. In typical growth, ∼1 g of lump VO_2_ (obtained by reducing V_2_O_5_ in a N_2_ atmosphere) and 8 g of V_2_O_5_ powder are introduced into a silica tube. The tube is heated up to about 1050 ^∘^C and then slowly cooled down to 775 ^∘^C with a cooling rate of ∼3 ^∘^C/h. Titanium powder in different quantities was added into the VO_2_–V_2_O_5_ mixture before annealing, and its concentration was determined in the high–temperature melt via wave–dispersive spectroscopy [18]. The crystal quality of the samples was assessed by Kong et al. through the X–ray diffraction technique [18]. Three samples of Ti-doped VO_2_ single crystal were prepared (Ti concentration x = 0.059, x = 0.082, and x = 0.187), with dimensions of 5 mm along the growth axis, and 500 µm × 500 µm wide on the orthogonal plane. In this work, we will refer to the long growth axis of the crystal as ${\vec{c}}_{R}$, as it is the c axis of the rutile phase (R).

**Figure 1:**
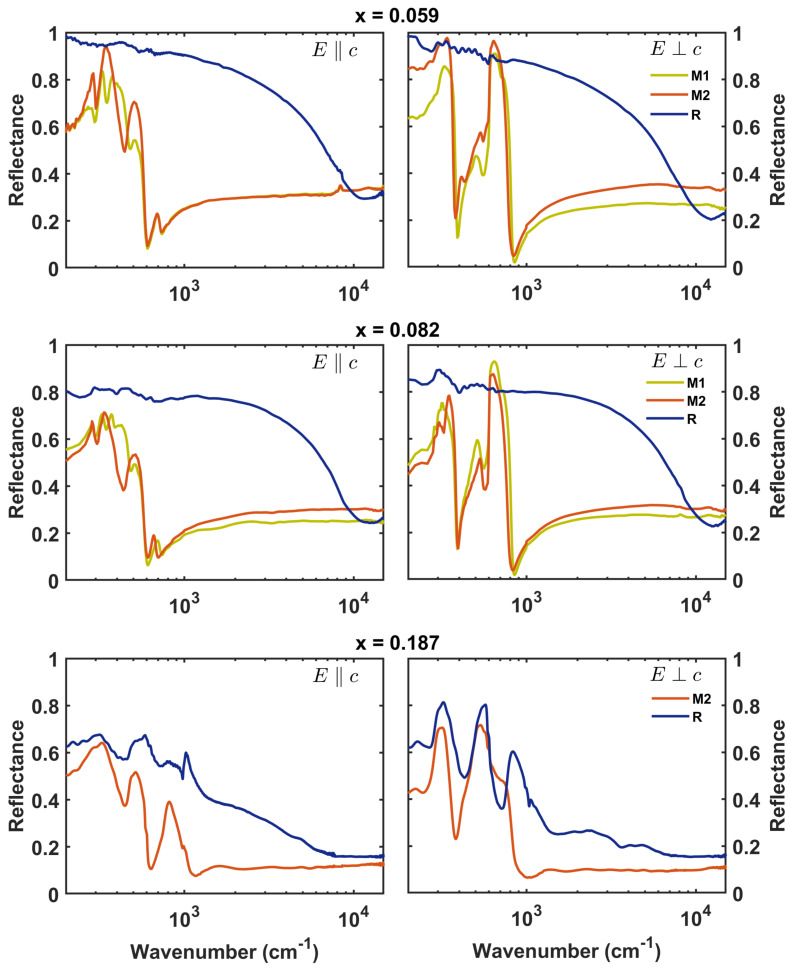
Reflectance spectra obtained from polarized measurements on single crystals with different Ti dopings from x = 0.059 to x = 0.187. The left column shows measurements with polarization parallel (perpendicular) to the growth axis, C_*R*_, and the right column shows measurements with polarization perpendicular to that. It is noticeable that the reflectivity of the rutile phase (blue lines) decreases as the doping increases (see text). As discussed previously, the monoclinic M1 phase is absent in the sample with x = 0.187.

While pure VO_2_ has a tetragonal rutile (P4_2_/mnm) structure labeled R in the metallic phase and an insulating monoclinic M1 (P2_1_/c) structure in the low–temperature insulating phase, Ti–doped samples can present also a secondary monoclinic structure (C2/m) called M2, which has half of the V–V pairs dimerized and half of them tilted along the direction [7,18]. Samples with x = 0.059 and x = 0.082 are in the M1 phase at room temperature, pass through the M2 phase, and reach the metallic R phase as the temperature increases. In contrast, samples with x = 0.187 do not show the M1 phase (V–V bond formation is hindered by the presence of the high concentration of titanium) and go from the M2 phase to the metallic R phase directly [18]. The surfaces of all samples were polished with diamond powder polishing suspension down to a surface roughness of about 300 nm for the reflectivity measurements. To study the infrared optical properties of these samples, polarized reflectivity measurements were performed at the SISSI beamline [21] of the Elettra synchrotron facility, in the spectral range between 200 cm^−1^ and 15,000 cm^−1^, with a resolution of 4 cm^−1^. Spectra were collected by probing in a direction parallel ($\|{\vec{c}}_{R}$) and perpendicular ($⊥{\vec{c}}_{R}$) to the long axis of each crystal via a Vertex 70v interferometer (Bruker) coupled with a Hyperion 2000 microscope. Two detectors were employed to cover this frequency range, a liquid-nitrogen-cooled MCT from 500 cm^−1^ to 15,000 cm^−1^ and a liquid-helium-cooled bolometer from 200 cm^−1^ to 500 cm^−1^. Temperature control of the sample was performed with a THMS350 Linkam stage, allowing the measurement of all the samples at 300 K for the “low temperature” insulating phase and at 372 K for the “high temperature” conductive rutile phase. The high temperature was set above the highest $T_{C}$∼365 K of the doped samples, which is the one with x = 0.187 doping [18]. Reflectance spectra, R(ω), of the samples, showed in Figure 1, were obtained from the ratio between the intensity reflected by the sample I_*R*_ (*ω*) and the intensity hitting the sample, I_0_(*ω*). The reflectivity data were analyzed with RefFIT software (Version 1.3.05), which allowed for the extraction of the optical properties of each sample in a form due to the Drude–Lorentz semiclassical approach, with the help of a Kramers–Kronig constrained variational method [22].

## 3. Results

### 3.1. Metallic Phase

**Figure 2:**
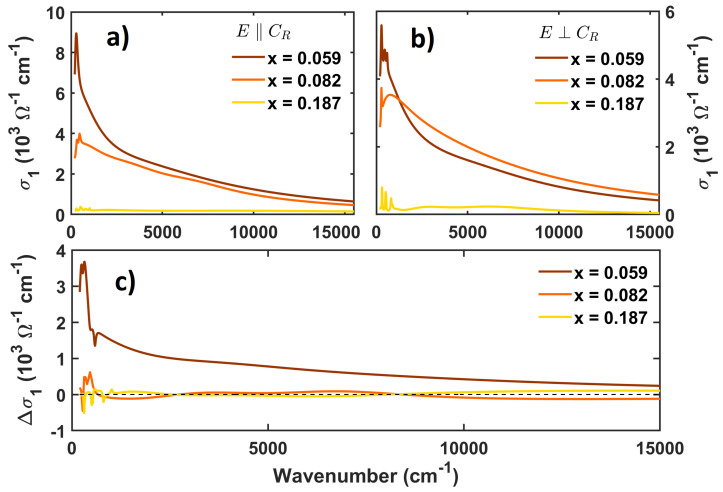
Real part of the optical conductivity of Ti_*x*_V_1−*x*_O_2_ single crystals in the R phase obtained with polarization (**a**) parallel and (**b**) orthogonal to the growth axis, $c_{R}$. Brown, orange, and yellow lines denote Ti doping with x = 0.059, x = 0.082, and x = 0.187, respectively. (**c**) Difference between the real part of the optical conductivities obtained with parallel and orthogonal polarization under fixed doping, as defined in Equation (1).

Figure 1 displays the different reflectance features as a function of Ti doping and the crystal phase. The left column of the figure is related to measures with polarization parallel to the growth axis, $C_{R}$, and the right column is related to measures with polarization perpendicular to that. The three different crystal phases are highlighted in different–colored lines: the blue line is for the conductive rutile (R) phase; the yellow line is for the first monoclinic phase, M1; and the second monoclinic phase, M2, is in orange. In all the panels, we see a strong difference in the reflectance shape between the rutile phase and the monoclinic ones due to the conductivity change. As expected, the conductive metallic rutile phase has a higher reflectance, with a characteristic Drude feature, while in the insulating monoclinic phases, we can more clearly observe the phonon features that are not screened by the Drude contribution. From the fitting process, a set of Drude–Lorentz oscillators was extracted from each reflectance spectrum. Each set allows us to determine the optical conductivity, $\tilde{\sigma }\left(\omega \right)$, or dielectric function, $\tilde{\epsilon }\left(\omega \right)$, as a function of the doping *x*, crystal structure, and polarization direction. In Figure 2a,b, the real part of the optical conductivity is shown for the crystals in the metallic phase above T_C_. In each panel, the real part of the optical conductivity, $\sigma_{1}\left(\omega \right)$, is shown for increasing doping and fixed polarization conditions.

In titanium–doped metallic VO_2_, along the ${\vec{c}}_{R}$ axis, Ti atoms elongate the V–V bonds and locally distort the VO_6_ octahedra [19]. This distortion propagates to chains without Ti ions, generating a general reduction in metallicity. Indeed, in Figure 2, we report a strong decrease in the low–frequency $\sigma_{1}\left(\omega \right)$ of the metallic phase with increasing titanium substitution. We also remark upon the clear measurable anisotropy among the conductivities for $\vec{E}\|{\vec{c}}_{R}$ and $\vec{E}⊥{\vec{c}}_{R}$ at a fixed x. This behavior is also observed in pure samples [3,23] and is consistent with previously reported values of the DC conductivity in pure single crystals of VO_2_ [9]. D’Elia et al. [23] observed that the band anisotropy in VO_2_ is also reflected in the conductive properties. The screening length difference between π* and $d_{\left|\right|}$ bands generates strongly anisotropic screening capabilities in VO_2_ and hence anisotropy in the conductivity. To stress this result, the difference between the optical conductivity for the two polarizations, which is defined as

(1) $$\Delta \sigma_{1}\left(\omega ,x\right)=\sigma_{1}\left(\omega ,\vec{E}\|{\vec{c}}_{R},x\right)-\sigma_{1}\left(\omega ,\vec{E}⊥{\vec{c}}_{R},x\right)$$

is shown in panel (c) of Figure 2.

While $\Delta \sigma_{1}\left(\omega ,x\right)$ is ∼0 for x = 0.082 and x = 0.187, it shows a strong difference, of up to 3000 cm^−1^, for x = 0.059. Thus, for low titanium content, we can see strong optical anisotropy similar to that of the undoped VO_2_, while this anisotropic response is strongly reduced under higher Ti doping. Table 1 lists the squared plasma frequencies, $\omega_{P}^{2}$, as a function of doping, obtained from the fitting procedure. Wu et al. [24] have shown that in a Ti_*x*_$V_{1-x}$O_2_ system, the carrier concentration does not experience a significant change. From the analysis of the sperctra, we extracted the plasma frequency characterized by the Drude metallic behavior of Ti_*x*_$V_{1-x}$O_2_ samples. Its square value is proportional to the carrier density and effective mass ratio. Therefore, the plasma frequency decrease, in view of Ref. [24], could be associated with an increase in the carriers’ effective mass, rather than a decrease in the carrier density, probably due to the lattice distortion local structure perturbations induced by Ti dopants.

### 3.2. Insulating Phases

**Figure 3:**
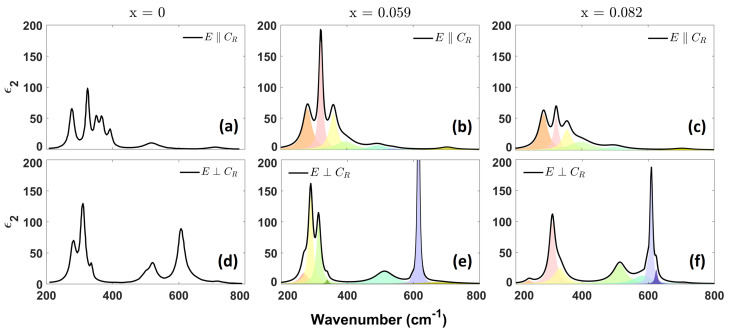
Imaginary part of the dielectric function of Ti_*x*_V_1−*x*_O_2_ single crystals in the M1 phase, with polarization (**a**–**c**) parallel and (**d**–**f**) orthogonal to the growth axis, ${\vec{c}}_{R}$. Each black line denotes the total $\epsilon_{2}\left(\omega \right)$, while colored sections represent single phononic modes. $\epsilon_{2}\left(\omega \right)$ presented in panels (**a**,**d**) are included for samples with x = 0 obtained from reference [9].

**Figure 4:**
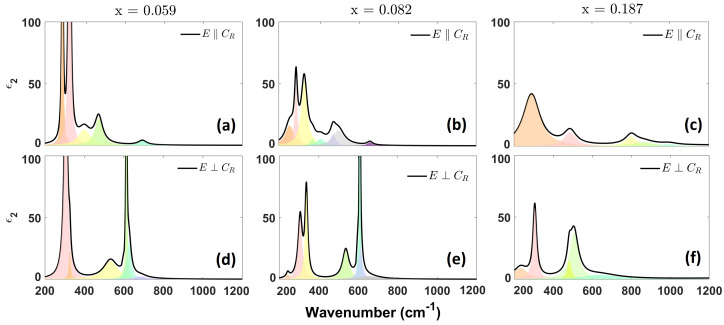
Imaginary part of the dielectric function of Ti_*x*_V_1−*x*_O_2_ single crystals in the M2 phase, with polarization (**a**–**c**) parallel and (**d**–**f**) orthogonal to the growth axis. Each black line denotes the total $\epsilon_{2}\left(\omega \right)$, while colored areas represent single phononic modes. The vertical scale is set to 100 cm^−1^ to better show the low-intensity phonons.

The imaginary part of the permittivity, $\epsilon_{2}\left(\omega \right)$, of the insulating phases below T_*C*_ are presented below. In Figure 3 and Figure 4, we show the phonon region of the $\epsilon_{2}\left(\omega \right)$ spectra for the insulating M1 and M2 phases, respectively.

The effect of titanium substitution in the insulating phases of VO_2_ results in a distortion of the monoclinic structure, which is perceived only through V–V pairs along the same chain [19].

Group theory applied to monoclinic M1 VO_2_ shows that there are eight infrared-active phonons for polarization parallel to the growth axis (the ${\vec{c}}_{R}$ axis in the rutile phase, and b in the M1 phase) and seven infrared-active modes for polarization in the plane perpendicular to it. Generally speaking, the peaks in the range 180 ÷ 250 cm^−1^ are associated with V–V vibrations [3,9]. The group at 250 ÷ 700 cm^−1^ is associated with V–O modes; those at about 280 and 500 cm^−1^ are related to a combination of O–V–O bending and O–V stretching vibrations, while those at 360–400 cm^−1^ are related only to O–V–O bending, and those at 690 cm^−1^ show pure V–O stretching modes [8].

#### 3.2.1. M1 Insulating Phase

In this section, we focus on the first monoclinic phase, M1, present in two doped samples and the undoped VO_2_. The imaginary part of the dielectric function, $\epsilon_{2}\left(\omega \right)$, for different Ti concentrations, is shown in Figure 3. The six panels are arranged so that each column is related to a different doping, *x*, value, while the two rows are associated with the electric field polarization orientation. To better compare the phonon mode evolution, in the first column (x = 0), the polarized $\epsilon_{2}\left(\omega \right)$ spectra for a pure VO_2_ crystal obtained by Huffman et al. [9] are shown. Phononic frequencies are listed in Table 2 and Table 3 for parallel and orthogonal polarization, respectively.

Comparing the phonon frequencies, we find that the main peaks do not have strong shifts for increased doping, for both polarizations, and many of the peaks are within the measurement error. Some broadening and reduction in intensity can be seen at frequencies 350–400 cm^−1^ for polarization $\|{\vec{c}}_{R}$, and around 300 cm^−1^ for polarization $⊥{\vec{c}}_{R}$.

#### 3.2.2. M2 Phase

The imaginary part of the dielectric function as a function of frequency, $\epsilon_{2}\left(\omega \right)$, with polarization parallel to the growth axis of the crystals, is shown in Figure 4a–c with different doping conditions. The group at frequencies 250 ÷ 400 cm^−1^ has an intensity that is drastically reduced from x = 0.059 to x = 0.082 and x = 0.187. The peak at about 700 cm^−1^ almost disappears from x = 0.082 to x = 0.187, and we observe the merging and broadening of the peaks at about 300 cm^−1^ at x = 0.187. We remark upon the appearance of a group of phonons at 800 ÷ 1000 cm^−1^ in the sample with x = 0.187, possibly due to clusters caused by the presence of titanium.

In Figure 4d–f, which show $\epsilon_{2}\left(\omega \right)$ for the M2 phase, with polarization orthogonal to the growth axis, we observe that each spectrum is composed of two bands recognizable for all *x* values, between 250 and 350 cm^−1^ and between 400 and 700 cm^−1^. In the first band, we observe splitting at x = 0.082 that disappears as titanium doping increases. The second band preserves its overall shape for x = 0.082, while on the other hand it broadens at x = 0.187.

In Table 4 and Table 5, the phonon frequencies for parallel and orthogonal polarization, respectively, are listed.

With increasing doping, we observed a general broadening of almost all the peaks. Moreover, we observed that the damping due to increasing doping was substantially stronger for $\vec{E}\|{\vec{c}}_{R}$; thus, we remark on the fact that titanium substitution is more effective along this axis.

## 4. Conclusions

We performed polarized infrared micro–spectroscopy on titanium–doped vanadium dioxide. For the first time, we obtained the phononic and electronic response in the M1, M2, and R phases of single-domain samples of Ti_*x*_$V_{1-x}$O_2_ from the far–infrared to the visible region The electronic part of the infrared optical conductivity of metallic Ti-doped VO_2_ is anisotropic and was found to be higher along the C_*R*_ axis compared with that on the orthogonal plane. The dielectric permittivity plots were extracted from the fitting of the reflectance spectra, allowing an analysis of the IR phonon modes for increasing doping. These plots show a general progressive broadening of and reduction in the intensity of almost all modes and the appearance of modes not belonging to VO_2_ or TiO_2_ [25]. This effect was more noticeable when the samples were in the M2 phase, when the concentration of Ti was higher, and for polarization parallel to the growth axis of the crystals. All these effects are traceable to the lattice distortion induced by the relative size of the dopant ion, Ti, which is larger compared with that of the vanadium one [24]. These results show that Ti doping offers a promising avenue for tailoring the electronic and thermochromic characteristics of VO_2_, opening up new possibilities for its utilization in advanced technologies.

## Figures and Tables

**Table 1:** Squared plasma frequencies, as a function of doping, *x*.

	$\omega_{P}^{2}$$\|{\vec{c}}_{R}$ ($10^{8}$ cm^−2^)	$\omega_{P}^{2}$$⊥{\vec{c}}_{R}$ ($10^{8}$ cm^−2^)
x = 0.059	5.8	2.8
x = 0.082	2.5	1.9
x = 0.187	0.9	0.04

**Table 2:** Phononic central frequencies, $\omega_{0}$ (in cm^−1^), for undoped (x = 0) single crystals [9] and for our Ti-doped single crystals (x = 0.059, x = 0.082), in the monoclinic M1 phase, with parallel polarization.

x = 0	277	324	351	367	392		519	709
x = 0.059	281	321	360		397	494	539	703
x = 0.082	285	323	356		399	501		704

**Table 3:** Phononic central frequencies, $\omega_{0}$ (in cm^−1^), for undoped (x = 0) single crystals [9] and for our Ti-doped single crystals (x = 0.059, x = 0.082), in the monoclinic, M1, phase, with orthogonal polarization.

x = 0		281	310	336	500	521		607	637	720
x = 0.059	268	289	313	339		513		615		673
x = 0.082	241		310	333		514	584	609	624	708

**Table 4:** Phononic central frequencies, $\omega_{0}$ (in cm^−1^), for our Ti–doped single crystals (x = 0.059, x = 0.082, x = 0.0187), in the monoclinic M2 phase, with parallel polarization.

x = 0.059		289	314	325		401	472		694			
x = 0.082	256	287		329	371	413	475	503	661			
x = 0.187		295						489		802	866	998

**Table 5:** Phononic central frequencies, $\omega_{0}$ (in cm^−1^), for our Ti–doped single crystals (x = 0.059, x = 0.082, and x = 0.0187), in the monoclinic M2 phase, with orthogonal polarization.

x = 0.059		293		420			529	583	607	
x = 0.082	241	306	337				537		608	649
x = 0.187	233	304		451	480	506				650

## Data Availability

The original contributions presented in the study are included in the article, further inquiries can be directed to the corresponding author.
